# Predictive biological factors for late survival in patients with HER2-positive breast cancer

**DOI:** 10.1038/s41598-023-38200-y

**Published:** 2023-07-07

**Authors:** Young-Joon Kang, Se Jeong Oh, Soo Youn Bae, Eun-Kyu Kim, Young-Jin Lee, Eun Hwa Park, Joon Jeong, Heung Kyu Park, Young Jin Suh, Yong-Seok Kim

**Affiliations:** 1 Department of Surgery, College of Medicine, Incheon St. Mary’s Hospital, The Catholic University of Korea, Incheon, Republic of Korea; 2 Department of Surgery, College of Medicine, Seoul St. Mary’s Hospital, The Catholic University of Korea, Seoul, Republic of Korea; 3 Department of Surgery, Seoul National University College of Medicine, Breast Care Center, Seoul National University Bundang Hospital, Seoul, Republic of Korea; 4 Division of Breast Surgery, Department of Surgery, Asan Medical Center, University of Ulsan College of Medicine, Seoul, Republic of Korea; 5 Department of Surgery, Dong-A University College of Medicine, Busan, South Korea; 6 Department of Surgery, Gangnam Severance Hospital, Yonsei University College of Medicine, Seoul, Republic of Korea; 7 Department of Surgery Breast Cancer Center, Gil Medical Center of Gachon University, Incheon, Republic of Korea; 8 Division of Breast and Thyroid Surgical Oncology, Department of Surgery, College of Medicine, St. Vincent’s Hospital, The Catholic University of Korea, Suwon, Republic of Korea; 9 Department of Surgery, College of Medicine, Uijeongbu St. Mary’s Hospital, The Catholic University of Korea, 271, Cheonbo-ro, Uijeongbu-si, Gyeonggi-do 11765 Republic of Korea

**Keywords:** Breast cancer, Breast cancer

## Abstract

The human epidermal growth factor receptor-2 (HER2) enriched subtype of breast cancer is associated with early recurrence, mostly within 5 years. However, anti-HER2 therapies have improved outcomes and their benefits persist in the long term. This study aimed to determine predictive factors for late survival in patients with HER2-positive breast cancer. We analyzed 20,672 patients with HER2-positive stage I–III breast cancer. The patients were divided into two groups based on a follow-up period of 60 months. The multivariate analysis of factors associated with poor overall survival included old age, advanced pathologic tumor size stage (pT), advanced pathologic regional lymph node stage (pN), high histological grade, presence of lymphatic and vascular invasion, and HR-negative status within 60 months. In the breast cancer-specific survival (BCSS) of the > 60 months follow-up group, the hazard ratios (HRa) based on pN-negative were 3.038, 3.722, and 4.877 in pN1 (*p* = 0.001), pN2 (*p* < 0.001), and pN3 (*p* < 0.001), respectively. Only pT4 level was statistically significant in the pT group (HRa, 4.528; *p* = 0.007). Age (HRa, 1.045, *p* < 0.001) and hormone receptor-positive status (HRa, 1.705, *p* = 0.022) were also associated to worse BCSS. Although lymphatic invasion was not significantly associated with BCSS, there was a tendency toward a relationship (*p* = 0.079) with worse BCSS. In HER2-positive breast cancer patients, node status had a more significant relationship with long-term prognosis than T stage. Patients with HER2-positive breast cancer who have T4 or node-positive should be considered for clinical observation and education beyond 5 years.

## Introduction

Breast cancer with amplification or overexpression of human epidermal growth factor receptor 2 (HER2) accounts for approximately 15% of primary invasive diseases^1^. HER2 positivity is associated with high recurrence and mortality^2^. In addition, HER2-positive breast cancers have a higher propensity to metastasize to the brain^3^. However, the advent and implementation of new anti-HER2 therapies have dramatically changed the treatment of patients with HER2 positivity^4^.

Significant progress has been made in treating patients with HER2-positive breast cancer, and studies are underway on the continuous improvement of outcomes in a subset of these patients. Nevertheless, patients treated for breast cancer remain at risk of recurrence^5^. There are many cases of late recurrence of hormone receptor (HR)-positive breast cancer, occurring even after 5 years^6,7^. Therefore, most studies have focused on HR-positive HER2-negative breast cancer. The HER2-enriched and basal subtypes are mostly associated with early recurrence within 5 years^8^. Recent advancements in treatment have improved the outcomes of patients with HER2-positive breast cancer. The survival rate at 4 years among women was 90.3% for HR-positive/HER2-positive breast cancer and 82.7% for HR-negative/HER2-positive breast cancer in 2018^9^.

Breast cancer has poor prognosis to be high nuclear grade, HR-negative, and HER2-positive, have a high proliferation fraction, present with lymphovascular invasion (LVI), and be diagnosed at young age and at more advanced stage^10–12^. Clinical factors that affect the prognosis of HER2-positive breast cancer have been studied. HR-positive/HER2-positive breast cancers are associated with better survival rates than HR-negative/HER2-positive breast cancers^13^.

Despite improvements in both disease-free survival and overall survival (OS) in early-stage HER2-positive breast cancer, long-term follow-up results indicate that approximately 15–24% of patients still develop recurrent disease^14,15^. Few studies have examined the long-term prognostic factors for nonmetastatic HER2-positive breast cancer. We aimed to identify factors associated with the long-term prognosis of patients with HER2-positive breast cancer.

## Results

### Patient demographics and clinicopathological characteristics

**Figure 1:**
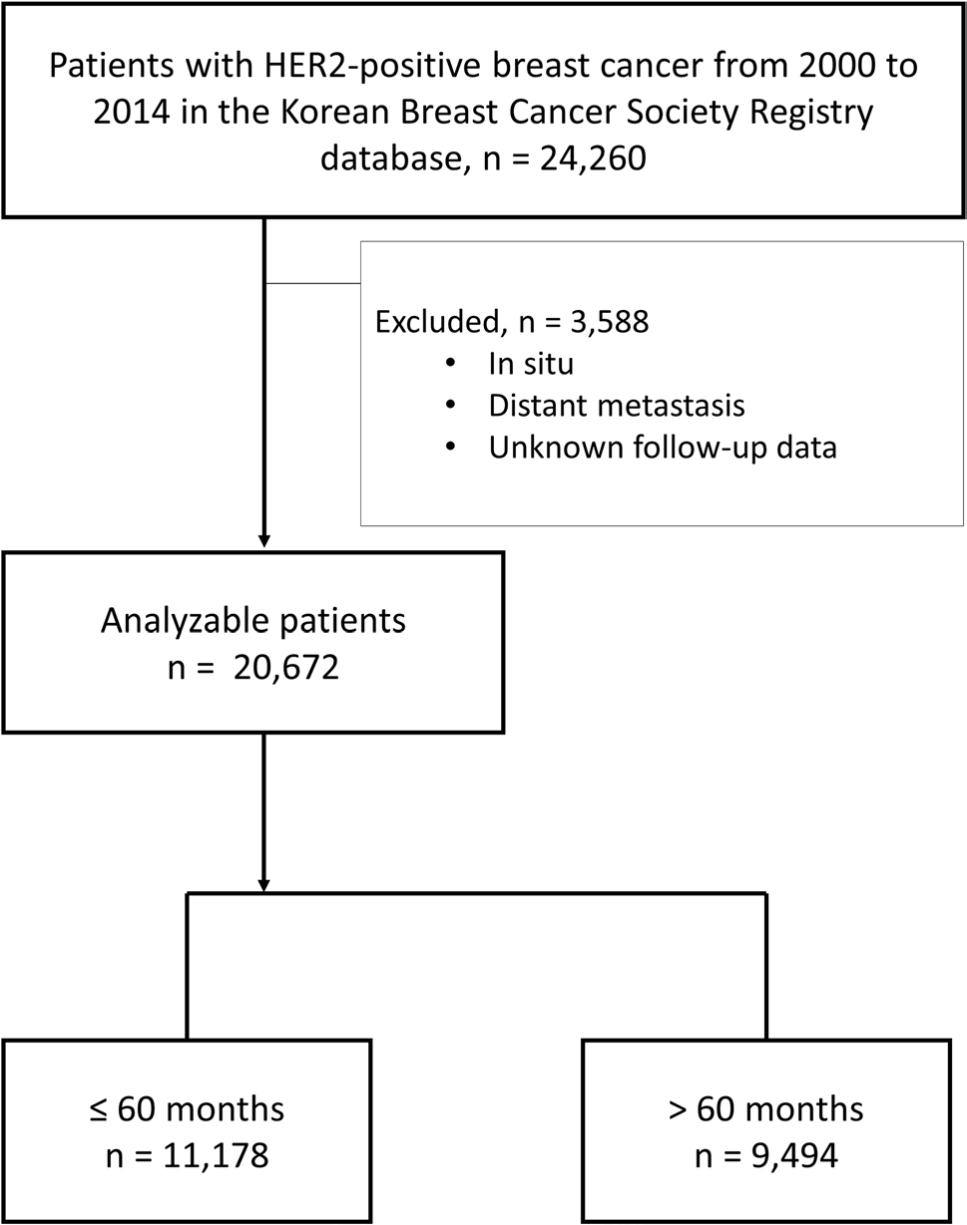
Flow of patients through the study.

A total of 24,260 patients were identified as candidates for inclusion in the analysis. Of these patients, 3161 and 425 had in situ and distant metastases, respectively, and were excluded from this study. Finally, 20,672 patients were analyzed after excluding two patients with unknown follow-up data (Fig. 1). The ≤ 60 months and > 60 months follow-up groups had median follow-up times of 29.4 and 97.9 months, respectively. Most clinicopathological characteristics were significantly different between the groups (Table 1). The mean age was 51.4 years in the ≤ 60 months follow-up group and 49.5 years in the > 60 months follow-up group. There was no significant difference in body mass index between the two groups (23.66 and 23.57, respectively). The groups had significantly different pathologic tumor size stage (pT; *p* < 0.001) and pathologic regional lymph node stage (pN; *p* < 0.001), however, the distribution in each stage was not significantly different. Other pathological characteristics analyzed, namely, histological grade, estrogen and progesterone receptor status, ki-67 index, p53 overexpression, and the presence of lymphatic and vascular invasion, are presented in Table 1. The therapeutic characteristics were also significantly different between the two groups. In the ≤ 60 months follow-up group, the rate of breast-conserving surgery was higher than that in the > 60 months follow-up group (47.6% and 37.1%, respectively; *p* < 0.001), and the rate of sentinel lymph node biopsy was higher (46.5% and 10.2%, respectively; *p*  <  0.001). In addition, the axillary lymph node dissection rates in the two groups were 51.2% and 81.4%, respectively (*p* <  0.001). Radiotherapy was performed more often in the ≤ 60 months follow-up group (62.8% vs. 50.4%; *p* < 0.001). In both groups, > 80% of patients received systemic chemotherapy (80.3% and 83.0%, respectively). No clear records were found for the 15,416 patients who underwent anti-HER2 therapy. Data on anti-HER2 therapy were missing for 6524 and 8892 patients in the ≤ 60- and > 60 months follow-up groups, respectively. A total of 539 and 157 patients in the ≤ 60- and > 60 months follow-up groups died from breast cancer, corresponding to rates of 4.8% and 1.7%, respectively.

**Table 1:** Patient characteristics according to follow-up period.

	Follow-up period		Total	*p* value
	≤ 60 months (n = 11,178)	> 60 months (n = 9494)	(n = 20,672)	
Follow-up periods (months)				< 0.001
Number	11,178	9494	20,672	
Mean	29	98	61	
Range	0–60	61–179	0–179	
Age (years)				< 0.001
Number	11,044	9493	20,537	
Mean	51.4	49.5	50.5	
Range	21–99	21–90	21–99	
Unknown	134	1	135	
BMI				0.056
Number	9046	8000	17046	
Mean	23.66	23.57	23.62	
Range	14.45–42.29	12.84–72.13	12.84–72.13	
Unknown	2132	1494	3626	
pT stage				< 0.001
pT1	6143 (56.0%)	4993 (53.2%)	11,136 (54.7%)	
pT2	4121 (37.6%)	3850 (41.0%)	7971 (39.2%)	
pT3	531 (4.8%)	447 (4.8%)	978 (4.8%)	
pT4	178 (1.6%)	93 (1.0%)	271 (1.3%)	
Unknown	205	111	316	
pN stage				< 0.001
pN0	6554 (59.1%)	5726 (60.7%)	12,280 (59.8%)	
pN1	2703 (24.4%)	2352 (24.9%)	5055 (24.6%)	
pN2	1038 (9.4%)	877 (9.3%)	1915 (9.3%)	
pN3	801 (7.2%)	485 (5.1%)	1286 (6.3%)	
Unknown	82	54	136	
Histologic grade				< 0.001
1	503 (5.0%)	600 (7.4%)	1103 (6.1%)	
2	4003 (40.0%)	3593 (44.5%)	7596 (42.0%)	
3	5498 (55.0%)	3888 (48.1%)	9386 (51.9%)	
Unknown	1174	1413	2587	
Estrogen receptor				0.001
Negative	5956 (53.3%)	5279 (55.7%)	11,235 (54.4%)	
Positive	5211 (46.7%)	4191 (44.3%)	9402 (45.6%)	
Unknown	11,167	9470	20,637	
Progesterone receptor				< 0.001
Negative	7127 (63.8%)	5278 (60.5%)	12,855 (62.3%)	
Positive	4037 (36.2%)	3733 (39.5%)	7770 (37.7%)	
Unknown	14	483	497	
Ki-67 index				< 0.001
< 20%	3011 (34.5%)	1554 (43.6%)	4565 (37.1%)	
≥ 20%	5720 (65.5%)	2011 (26.0%)	7731 (62.9%)	
Unknown	2447	5929		
p53 overexpression				0.001
Negative	3763 (41.3%)	3279 (43.9%)	7042 (42.5%)	
Positive	5353 (58.7%)	4190 (56.1%)	9543 (57.5%)	
Unknown	2062	2025	4087	
Lymphatic invasion				0.004
Negative	6119 (64.1%)	5062 (66.2%)	11,181 (65.1%)	
Positive	3420 (35.9%)	2581 (33.8%)	6001 (34.9%)	
Unknown	1639	1851	3490	
Vascular invasion				0.003
Negative	6848 (78.0%)	5683 (79.9%)	12,531 (78.8%)	
Positive	1935 (22.0%)	1431 (20.1%)	3366 (21.2%)	
Unknown	2395	2380	4775	
Breast operation				< 0.001
BCS	5287 (47.6%)	3458 (37.1%)	8745 (42.8%)	
Total mastectomy	5829 (52.4%)	5870 (62.9%)	11,699 (57.2%)	
Missing data	62	166	228	
Axilla operation				< 0.001
No	253 (2.3%)	791 (8.4%)	1,044 (5.1%)	
SLNB	5191 (46.5%)	954 (10.2%)	6145 (29.9%)	
SLNB + ALND	2410 (21.6%)	1669 (17.8%)	4079 (19.9%)	
ALND	3304 (29.6%)	5957 (63.6%)	9216 (45.1%)	
Missing data	20	123		
Radiotherap				< 0.001
No	3740 (37.2%)	4137 (49.6%)	7877 (42.8%)	
Yes	6311 (62.8%)	4203 (50.4%)	10,514 (57.2%)	
Missing data	1127	1154	2281	
Hormonal therapy				< 0.001
No	4837 (50.1%)	3693 (46.1%)	8530 (48.3%)	
Yes	4812 (49.9%)	4322 (53.9%)	9134 (51.7%)	
Missing data	1529	1479	3008	
Chemotherapy				< 0.001
No	2077 (19.7%)	1,486 (17.0%)	3,563 (18.5%)	
Yes	8,440 (80.3%)	7,260 (83.0%)	15,700 (81.5%)	
Missing data	661	748	1,409	
Anti-HER2 therapy				< 0.001
No	7 (0.2%)	0 (0.0%)	7 (0.1%)	
Yes	4647 (99.8%)	602 (100.0%)	5249 (99.9%)	
Missing data	6524	8892	15,416	
Mortality				< 0.001
Alive	9571 (85.6%)	7837 (82.5%)	17,408 (84.2%)	
d/t breast cancer	539 (4.8%)	157 (1.7%)	696 (3.4%)	
d/t other cause	1068 (9.6%)	1500 (15.8%)	2568 (12.4%)	

*BMI* body mass index, *pT* pathological tumor, *pN* pathological regional lymph nodes, *BCS* breast-conserving surgery, *SLNB* sentinel lymph node biopsy, *ALND* axillary lymph node dissection, *HER2* human epidermal growth factor receptor 2, *d/t* due to.

### Survival outcomes and associated factors

**Figure 2:**
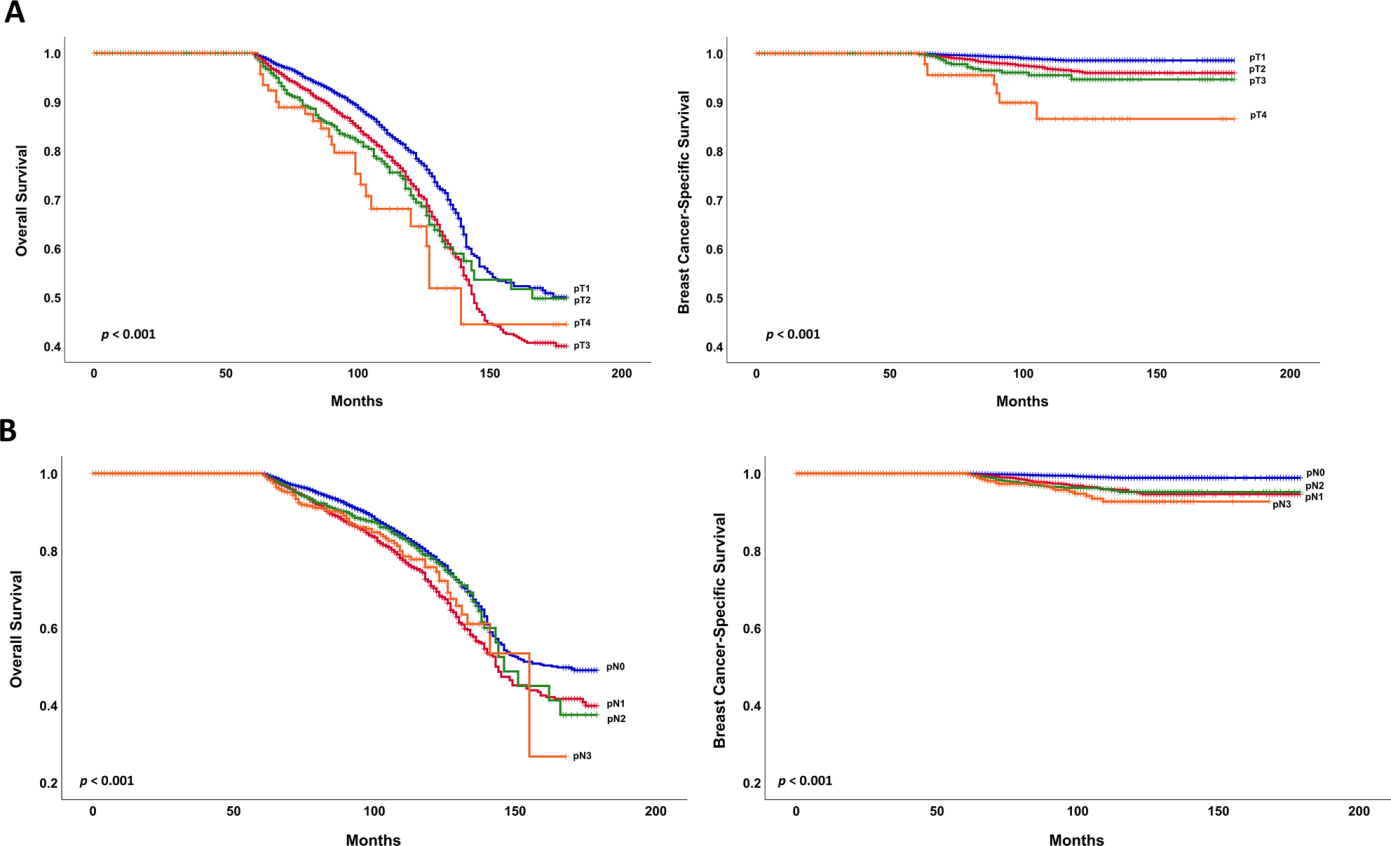
The Kaplan–Meier curves of overall survival and breast cancer-specific survival in over 60 months of follow-up (**A**) pT stage, (**B**) pN stage.

Univariate analysis was performed for the factors associated with breast cancer specific survival (BCSS). Old age, advanced pT and pN, elevated CEA levels, lymphatic and vascular invasion, and HR-negative status were statistically significant in both groups (Table 2). Multivariate analysis of factors associated with poor OS included old age, advanced pT, advanced pN, high histological grade, lymphatic and vascular invasion, and HR-negative status within 60 months (Table 3A). Advanced pT and pN, the presence of vascular invasion, and HR-positive status were associated with worse OS after 60 months (Table 3A). In the ≤ 60 months follow-up period of BCSS, poor outcome was associated with old age, advanced pT and pN stages, histological grade III, presence of lymphatic invasion, and HR-negative status. In the > 60 months follow-up period, the hazard ratios (HRa) based on pN0 were 3.038, 3.722, and 4.877 for pN1-3, indicating that pN stage was the most significant variable related to outcome (*p* = 0.001, *p*< 0.001, and *p* < 0.001, respectively). Only pT4 was significantly associated with worse BCSS than pT1 (reference level) in the > 60 months follow-up group (HRa, 4.528; *p* = 0.007). Old age (HRa, 1.045; 95% confidence interval (CI) 1.023–1.066; *p* < 0.001) and HR positivity (HRa, 1.705; 95% CI 1.079–2.963; *p* = 0.022) were also associated with poor outcomes. Although lymphatic invasion was not statistically significant, there was a tendency toward a *p-*value of 0.079 indicating a worse outcome (Table 3B). The Kaplan–Meier curve was used to analyze the pT and pN to compare the effect of OS and BCSS in the > 60 months follow-up period. The differences between the pT and pN stages were statistically significant (*p* < 0.001 for both; Fig. 2A,B). However, the patterns differed between the OS and BCSS. The Kaplan–Meier curve showed that pT4 was associated with significantly poorer BCSS than pT1-3. Regarding pN stage, pN0 was associated with better BCSS than pN1-3.

**Table 2:** Univariate analysis of factors for breast cancer-specific survival according to follow-up period.

		≤ 60 months		> 60 months	
		HRs (95% CI)	*p *value	HRs (95% CI)	*p *value
Age (years)	Per unit	1.020 (1.012–1.028)	< 0.001	1.033 (1.017–1.049)	< 0.001
Pathologic T stage	T1	1.000		1.000	
	T2	2.713 (2.210–3.331)	< 0.001	2.677 (1.856–3.862)	< 0.001
	T3	5.818 (4.367–7.752)	< 0.001	4.210 (2.367–7.487)	< 0.001
	T4	16.851 (12.185–23.304)	< 0.001	10.522 (4.940–22.412)	< 0.001
Pathologic N stage	N0	1.000		1.000	
	N1	3.629 (2.869–4.590)	< 0.001	4.337 (2.922–6.437)	< 0.001
	N2	6.617 (5.126–8.540)	< 0.001	4.871 (3.013–7.877)	< 0.001
	N3	11.342 (8.817–14.589)	< 0.001	7.127 (4.192–12.118)	< 0.001
BMI	< 25	1.000		1.000	
	≥ 25	1.231 (1.013–1.495)	0.036	1.188 (0.826–1.708)	0.354
CEA	Normal	1.000		1.000	
	Elevation	3.125 (2.100–4.651)	< 0.001	2.813 (1.113–7.111)	0.029
CA15-3	Normal	1.000		1.000	
	Elevation	2.974 (2.035–4.347)	< 0.001	1.228 (0.389–3.874)	0.726
Histologic Grade	1, 2	1.000		1.000	
	3	1.658 (1.376–1.997)	< 0.001	1.012 (0.721–1.421)	0.943
Lymphatic invasion	No	1.000		1.000	
	Yes	3.877 (3.158–4.759)	< 0.001	3.537 (2.368–5.285)	< 0.001
Vascular invasion	No	1.000		1.000	
	Yes	3.152 (2.574–3.860)	< 0.001	3.136 (2.097–4.688)	< 0.001
Hormone Receptor^a^	Negative	1.000		1.000	
	Positive	0.587 (0.493–1.699)	< 0.001	1.812 (1.303–2.520)	< 0.001
p53 overexpression	Negative	1.000		1.000	
	Positive	1.020 (0.839–1.240)	0.843	1.027 (0.713–1.481)	0.885
Ki-67	< 20%	1.000		1.000	
	≥ 20%	1.346 (0.984–1.842)	0.063	0.930 (0.512–1.690)	0.813

*CI* confidence interval, *T* tumor, *N* node, *HRs* hazard ratios, *BMI* body mass index, *CEA* carcinoembryonic antigen, *CA15-3* cancer antigen 15-3.

^a^Hormone Receptor was defined as positive when either estrogen receptor or progesterone receptor was positive.

**Table 3:** Multivariate Cox regression analysis of factors for survival according to follow-up period. A. Overall survival, B. Breast cancer-specific survival.

		≤ 60 months		> 60 months	
		HRs (95% CI)	*p* value	HRs (95% CI)	*p* value
A. Overall survival					
Age (years)*	Per unit	1.012 (1.006–1.018)	< 0.001	1.005 (0.998–1.012)	0.141
Pathologic T stage	Per stage				
T1		1.000		1.000	
T2		1.530 (1.316–1.778)	< 0.001	1.236 (1.067–1.431)	0.005
T3		2.223 (1.759–2.810)	< 0.001	1.550 (1.152–2.085)	0.004
T4		4.705 (3.508–6.311)	< 0.001	2.063 (1.192–3.569)	0.010
Pathologic N stage	Per stage				
N0		1.000		1.000	
N1		1.944 (1.629–2.321)	< 0.001	1.419 (1.205–1.671)	< 0.001
N2		2.740 (2.231–3.365)	< 0.001	0.939 (0.730–1.206)	0.621
N3		4.052 (3.293–4.987)	< 0.001	1.292 (0.961–1.737)	0.090
Grade	I, II/III	1.284 (1.124–1.466)	< 0.001	0.947 (0.824–1.089)	0.444
Lymphatic invasion	No/Yes	1.408 (1.183–1.674)	< 0.001	0.822 (0.678–0.997)	0.047
Vascular invasion	No/Yes	1.074 (0.911–1.267)	0.395	1.580 (1.294–1.928)	< 0.001
HR status	Negative/Positive	0.687 (0.602–0.783)	< 0.001	1.387 (1.205–1.597)	< 0.001
B. Breast cancer-specific survival					
Age (years)*	Per unit	1.029 (1.019–1.039)	< 0.001	1.045 (1.023–1.066)	< 0.001
Pathologic T stage	Per stage		< 0.001		
T1		1.000		1.000	
T2		1.550 (1.188–2.023)	< 0.001	1.480 (0.891–2.456)	0.130
T3		2.152 (1.444–3.207)	< 0.001	2.197 (0.980–4.924)	0.056
T4		5.746 (3.687–8.955)	< 0.001	4.528 (1.522–13.475)	0.007
Pathologic N stage	Per stage				
N0		1.000		1.000	
N1		2.459 (1.776–3.403)	< 0.001	3.038 (1.623–5.685)	0.001
N2		3.510 (2.431–5.068)	< 0.001	3.722 (1.831–7.564)	< 0.001
N3		6.107 (4.269–8.738)	< 0.001	4.877 (2.256–10.545)	< 0.001
Grade	I, II/III	1.320 (1.049–1.663)	0.018	1.193 (0.765–1.861)	0.435
Lymphatic invasion	No/Yes	1.384 (1.020–1.878)	0.037	1.708 (0.939–3.105)	0.079
Vascular invasion	No/Yes	1.307 (0.991–1.725)	0.058	1.379 (0.806–2.360)	0.240
HR status	Negative/Positive	0.654 (0.521–0.820)	< 0.001	1.705 (1.079–2.693)	0.022

*HRs* hazard ratios, *CI* confidence interval, *T* tumor, *N* node, *HR* hormonal receptor.

*Continuous variables.

### Additional data and subgroup analysis

The factors for BCSS related to a follow-up period of > 60 months could not be identified by analyzing only the patients who received anti-HER2 therapy. Among 5249 patients who received anti-HER2 therapy, 15 died of breast cancer within 60 months of diagnosis (Supplement 1). Subgroup analyses were performed according to staging. In stage I, age was the only factor related to BCSS after 60 months, whereas age and lymphatic invasion were associated factors in stage II. No associated factors were identified in stage III patients (Supplement 2).

## Discussion

The advent of anti-HER2 therapy has dramatically improved the outcome of patients with HER2-positive breast cancer. Few studies have reported the factors associated with the long-term prognosis of patients with HER2-positive nonmetastatic breast cancer. Although it was difficult to analyze the long-term prognosis with data reflecting the rapidly changing trend of HER2-positive breast cancer therapy, it was possible to infer factors affecting breast cancer specifically in HER2-positive breast cancer, by comparing the OS with the same period. In this study, we analyzed 20,672 patients and demonstrated that closer follow-up of HER2-positive breast cancer patients might be required, even after 5 years in patients with T4 or node-positive breast cancer. Multivariate analysis and Kaplan–Meier survival curves indicated that pT4 or node positivity was also a significant factor in long-term prognosis. Additionally, in elderly individuals, HR positivity or lymphatic invasion may be associated with the prognosis of patients with HER2-positive breast cancer.

The characteristics between the two groups by follow-up period indicated similar distributions of each factor, notwithstanding a statistically significant divergence. In particular, although the pathological characteristics of the patients showed similar distribution patterns, the treatment factors adopted discrepancies between groups. The differences in treatments might be influenced by evolving trends in clinical practice, reflecting the more recent diagnosis of patients in the ≤ 60 months follow-up group. Specifically, this data showed that breast-conserving surgery was more prevalent by 10.5% in the ≤ 60 months follow-up group relative to the > 60 months follow-up group. Furthermore, the incidence of patients undergoing sentinel lymph node biopsy alone was considerably higher in the ≤ 60 months follow-up group, standing at a marked 36.3%. Radiotherapy was also more common in the ≤ 60 months group, with a 12.4% higher rate. These observed variations may be attributed to changing paradigms in breast cancer management, highlighting the gradual shift towards less aggressive and more optimized patient treatment modalities.

Lee and Kim analyzed 131,178 patients with nonmetastatic breast cancer between 1980 and 2014. The study showed that lymph node metastasis was associated with increased mortality in HER2-positive tumors compared to an increase in T stage^16^. This was observed in HER2-positive patients regardless of HR status. Other studies have also shown that HER2-positive breast cancer has a higher rate of lymph node metastasis than the other types^17–19^. The degree of lymphatic vessel density was significantly associated with breast cancer subtype, with the HER2 subtype showing the highest density of^20^. Similar results were observed in this study. Lymph node metastasis showed the most significant association with long-term prognosis, and lymphatic invasion showed a similar trend; however, the linear association with T stage was insignificant.

Another study reported that tumor size > 2 cm and positive node status, irrespective of subtype, affected breast cancer-related survival in long-term follow-up (median follow-up of 18.7 years)^21^. Our study only analyzed patients with HER2-positive diseases. Nodal positivity showed the same results in each analysis, but the tumor stage revealed a different result from that of BCSS. We showed that only the T4 stage was significantly associated with BCSS. T3 tended to be associated with BCSS (*p* = 0.056). There was a difference within 5% for T1 in the Kaplan–Meier curve, and there was no notable difference for T2. This finding indicates that tumor size is not related to poor long-term outcomes.

A distinct pattern of recurrence was observed according to HR status in HER2-positive disease for 5–10 years from NCCTG N9831 and NSABP B-31^22^. The benefit of adjuvant trastuzumab persisted in the long term, and the effect was similar in HR-positive and HR-negative, HER2-positive breast cancer patients^22^. We also found that HR status was a statistically significant factor in late prognosis. HR-negative status was related to poor survival outcomes within 60 months, whereas HR-positive status was associated with worse survival outcomes over 60 months of follow-up. The luminal type may show a recurrence pattern that can occur over a long period of time.

Neoadjuvant chemotherapy has become standard clinical practice and has increased the proportion of patients who receive neoadjuvant chemotherapy in recent years. In May 2022, the NCCN guidelines were updated to state that neoadjuvant systemic therapy can be considered for cT1c, cN0 HER2-positive disease^23^. A strong relationship between the pathological response and prognosis after neoadjuvant therapy has been reported^24,25^. Patients who achieved pathological complete response (pCR) had excellent long-term prognosis after neoadjuvant therapy^26^. In particular, the number of HER2-positive breast cancer patients who received neoadjuvant chemotherapy has increased because of the increased pCR rate resulting from trastuzumab plus pertuzumab^27^. Neoadjuvant chemotherapy with trastuzumab and pertuzumab increase the pCR rate by approximately 50–70%^27–29^. In addition, recurrence in patients with residual disease after neoadjuvant therapy improves with advanced adjuvant treatment^30^. Considering these current trends, when the data of patients treated with current advanced treatment were analyzed with respect to factors related to long-term prognosis, there is a possibility that these results would not agree with those based on data from patients treated earlier. Several studies have attempted to predict the prognosis of HER2-positive breast cancer patients after neoadjuvant therapy. The integration of tumor-infiltrating lymphocytes, circulating tumor cells, or circulating tumor DNA may enhance the prediction; however, for intuitive use in clinical practice, a more accessible factor is needed^31–33^. Therefore, it is necessary to identify clinical factors associated with long-term prognosis. Data from this study showed that systemic chemotherapy had no significant effect on the prognosis in the 60 months group. However, it should be taken into consideration that data were included from 2000, when systemic treatments, including anti-HER2 therapies, were different from current treatments.

There were several limitations in this study. The clinicopathological characteristics of patients with a follow-up time shorter than 60 months had a little representative and might cause bias in the results because of including the following situations. First, the patients had reached the clinical endpoint (death). Second, the follow-up time of some patients was short. The durations of follow-up were 0 to 60 months. Third, the rate of lost-to-follow-up was not analyzed in this data. However, the distribution of patients was inferred, and the flow of differences in the treatment was shown.

In addition, the important limitation was that data on death were recorded until 2014. Therefore, analyses of breast cancer mortality over 60 months of follow-up were more distributed in the 2000s, which is different from the current treatment. Considering the trends in systemic therapy, including the recent widespread use of anti-HER2 agents, the prognosis of these patients cannot be directly compared with that of patients currently being treated in 2022.

There are many missing data on anti-HER2 therapy. According to the National Health Insurance Service in Korea, considering the treatment policy, it is possible to assume that anti-HER2 therapy had been applied to most patients. Therefore, we attempted to re-analyze the patient data after 2008, assuming that they received trastuzumab treatment. However, data that did not provide information on anti-HER2 therapy were analyzed by treating them as missing data. There were several reasons for this: too much missing data for anti-HER2 treatment, survival data up to 2014, and T1a–b stage patients who were not treated with anti-HER2 therapy. In advanced breast cancer, anti-HER2 agents have doubled the median OS to > 50 months and have more than tripled the 5 years survival rate^34^. Therefore, anti-HER2 therapy, including trastuzumab, was predicted to be one of the most significant factors affecting long-term prognosis; however, this was excluded from the analysis. To complement this limitation, we compared OS and BCSS and identified that the higher the N stage in BCSS compared to OS, the more associated the long-term prognosis.

Another limitation was that missing Ki-67 data were found in 8376 (40.5%) cases, and available data were mostly included within the 5 years follow-up group (8731; 71% of the available Ki-67 data). Ki-67 has been shown to be a 10 years prognostic factor in HER2-positive or triple-negative breast cancer groups^35^. Therefore, the study was limited to analyzing long-term prognosis, and the Ki-67 index was excluded from the multivariate analysis. Neoadjuvant systemic therapy was not actively administered to the patients who received treatment between 2000 and 2014.

In conclusion, node status has a more significant relationship with long-term prognosis than T stage in patients with HER2-positive breast cancer. This finding indicates that tumor size itself is not related to poor outcomes in terms of long-term prognosis, and aggravation of nodal stage is associated with poor outcomes after 5 years. Additionally, close follow-up is required in elderly individuals and patients with lymphatic invasion or HR positivity. Although the evidence level is low, associated indications or guidelines for patients who require long-term observation and education may need to be established in the future.

## Material and methods

### Study population

This study used nationwide data from the Korean Breast Cancer Registry (KBCR; http://registry.kbcs.or.kr/ecrf). Since 1996, The Korean Breast Cancer Society has been prospectively collecting data from patients with breast cancer. The database provides demographic characteristics, patient history, clinicopathological characteristics, treatment modality information, and follow-up data. It was estimated that enrollment in 2013 included more than 65% of all newly diagnosed breast cancer patients in Korea^36^. The Korean Central Cancer Registry, Ministry of Health and Welfare, Korea, provided dates and causes of death on December 31, 2014. The KBCR database does not contain sufficient data regarding recurrence.

This study collected data from patients who underwent surgery for HER2-positive primary breast cancer between January 1, 2000, and December 31, 2014, in South Korea. Among the c-erbB2 ++ results, the patients who did not undergo in situ hybridization testing were excluded. The included patients were women aged > 18 years with pathological stage I–III disease. Patients were enrolled regardless of whether targeted therapy was administered. Patients who had received neoadjuvant systemic therapy or had a history of other cancers were also included. The pT was categorized as 1, 2, 3, and 4. The pN was divided into 0–3, and the micrometastatic lymph nodes were included in pN1. Institutions recorded HR status data as assessed by their analysis and cutoff values. HR positivity was defined as estrogen or progesterone receptor positivity. The cut-off value for the Ki-67 labeling index was set at 20%^37^. HER2-positive status was defined as an immunohistochemistry score of 3+ cell surface protein expression, or equivocal cases followed by a positive fluorescent or silver in situ hybridization test result according to the American Society of Clinical Oncology/College of American Pathologists HER2 testing guidelines (2007). Patients with no recorded HER2 status or equivocal status without in situ hybridization results were excluded. This study was approved by the Institutional Review Board of Incheon St. Mary’s Hospital (IRB number: OC22ZASI0020) and was conducted in accordance with the tenets of the Declaration of Helsinki. Informed consent was not obtained from any of the participants.

Patients were categorized according to the follow-up period. They were grouped into early and late prognosis groups, defined as having follow-up periods ≤ 60 and > 60 months, respectively. The chi-square test and Fisher’s exact test were used for categorical variables. Continuous variables were assessed using a t-test. This study aimed to determine predictive factors for late mortality in patients with HER2-positive breast cancer. Patients who died within 60 months were censored when analyzing the late mortality. The two groups were analyzed for OS and breast cancer-specific survival (BCSS). OS was defined as the interval from surgery to the date of death or last follow-up. BCSS was defined as survival until death due to breast cancer and censored by death from other causes. The Cox proportional hazard regression model was used for the univariate and multivariate survival analyses. Adjusted hazard ratios (HRa) with 95% confidence intervals (CIs) are reported. Statistical significance was set at *p* < 0.05. All statistical analyses were performed using Statistical Package for the Social Sciences, version 26.0 (IBM Corporation, Armonk, NY, USA).

### Ethical approval and consent to participate

The need of informed consent was waived by the Catholic University of Korea, Incheon St. Mary's Hospital Institutional Review Board (IRB no. OC22ZASI0020) of the Ethics Committee.

## Supplementary Information

Supplementary Tables.

## Data Availability

Data files are available from the Korean Breast Cancer Registry (KBCR; http://registry.kbcs.or.kr/ecrf). The datasets generated and/or analysed during the current study are not publicly available because the data is owned by the Korea Breast Cancer Society and is only available to those with permission among the society members; doctors associated with breast oncology. The data are available from the corresponding author on reasonable request.
