# Supplementary material for: Predictive biological factors for late survival in patients with HER2-positive breast cancer
Source: Sci Rep. 2023 Jul 7;13:11008. doi: 10.1038/s41598-023-38200-y (PMC10328940; doi:10.1038/s41598-023-38200-y)
Supplement: Supplementary file 1 — Supplementary Tables. [file 41598_2023_38200_MOESM1_ESM.docx]

| **Supplement 1**. Multivariate Cox regression analysis of factors for survival according to follow-up period in patients receiving anti-HER2 therapy (n=5,357) | | | | | | |
| --- | --- | --- | --- | --- | --- | --- |
| A. Overall survival |  |  |  |  |  |  |
|  |  | ≤ 60 months | |  | > 60 months | |
|  |  | HRs (95% CI) | *p-*value |  | HRs (95% CI) | *p-*value |
| Age (years)* | Per unit |  | 0.452 |  |  | 0.663 |
| pathologic T stage | Per stage |  |  |  |  |  |
| T1 |  | 1.000 |  |  | 1.000 |  |
| T2 |  | 1.157 (0.757-1.770) | 0.501 |  | 1.127 (0.274-4.641) | 0.869 |
| T3 |  | 1.518 (0.788-2.926) | 0.212 |  | 0.640 (0.061-6.666) | 0.709 |
| T4 |  | 4.395 (2.049-9.429) | <0.001 |  | 4.147 (0.401-42.914) | 0.233 |
| pathologic N stage | Per stage |  |  |  |  |  |
| N0 |  | 1.000 |  |  |  |  |
| N1 |  | 1.172 (0.668-2.057) | 0.581 |  | 2.296 (0.197-26.764) | 0.507 |
| N2 |  | 3.149 (1.761-5.632) | <0.001 |  | 4.743 (0.377-59.736) | 0.228 |
| N3 |  | 4.104 (2.306-7.304) | <0.001 |  | 15.560 (1.562-154.994) | 0.019 |
| Grade | I, II/III | 1.123 (0.763-1.654) | 0.556 |  | 1.467 (0.380-5.659) | 0.578 |
| Lymphatic invasion | No/Yes | 1.689 (1.033-2.761) | 0.037 |  | 3.120 (0.492-19.795) | 0.227 |
| Vascular invasion | No/Yes | 0.913 (0.577-1.445) | 0.697 |  | 1.116 (0.282-4.414) | 0.876 |
| HR status | Negative/Positive | 0.497 (0.334-0.739) | 0.001 |  | 0.232 (0.049-1.107) | 0.067 |
| B. Breast cancer-specific survival | |  |  |  |  |  |
|  |  | ≤ 60 months | |  | > 60 months† | |
|  |  | HRs (95% CI) | *p-*value |  | HRs (95% CI) | *p-*value |
| Age (years)* | Per unit | 0.952 (0.890-1.017) | 0.146 |  | NA |  |
| pathologic T stage | Per stage |  |  |  |  |  |
| T1 |  | 1.000 |  |  | 1.000 |  |
| T2 |  | 1.805 (0.457-7.136) | 0.400 |  | NA |  |
| T3 |  | 2.898 (0.449-18.713) | 0.264 |  | NA |  |
| T4 |  | NA |  |  | NA |  |
| pathologic N stage | Per stage |  |  |  |  |  |
| N0 |  | 1.000 |  |  | 1.000 |  |
| N1 |  | 1.285 (0.274-6.034) | 0.751 |  | NA |  |
| N2 |  | 0.693 (0.063-7.577) | 0.764 |  | NA |  |
| N3 |  | 2.381 (0.423-130412) | 0.325 |  | NA |  |
| Grade | I, II/III | 1.734 (0.535-5.618) | 0.359 |  | NA |  |
| Lymphatic invasion | No/Yes | 2.266 (0.600-8.555) | 0.227 |  | NA |  |
| Vascular invasion | No/Yes | 0.773 (0.199-3.004) | 0.711 |  | NA |  |
| HR status | Negative/Positive | 1.150 (0.392-3.372) | 0.799 |  | NA |  |
| HRs; hazard ratios, CI; confidence interval, T; tumor, N; node, HR: hormonal receptor, NA: not available | | | | | |  |
| *Continuous variables | |  |  |  |  |  |
| ^†^No data on breast cancer-specific death in the over 60 months of follow-up | | | | |  |  |

| **Supplement 2**. Subgroup analysis of factors related to survival over 60 months by stage | | | |  |
| --- | --- | --- | --- | --- |
| A. Overall survival |  |  |  |  |
| Stage | Factors |  | HRs (95% CI) | *p-*value |
| Stage I | Age (years)* | Per unit | 1.013 (0.999-1.026) | 0.060 |
|  | Grade | I, II/III | 1.021 (0.777-1.343) | 0.879 |
|  | Lymphatic invasion | No/Yes | 0.477 (0.272-0.836) | 0.010 |
|  | Vascular invasion | No/Yes | 1.801 (0.972-3.336) | 0.062 |
|  | HR status | Negative/Positive | 1.578 (1.209-2.061) | 0.001 |
| Stage II | Age (years)* | Per unit | 1.008 (0.999-1.018) | 0.082 |
|  | Grade | I, II/III | 0.936 (0.773-1.133) | 0.498 |
|  | Lymphatic invasion | No/Yes | 0.881 (0.686-1.133) | 0.324 |
|  | Vascular invasion | No/Yes | 1.719 (1.314-2.249) | <0.001 |
|  | HR status | Negative/Positive | 1.370 (1.127-1.666) | 0.002 |
| Stage III | Age (years)* | Per unit | 0.982 (0.966-0.998) | 0.027 |
|  | Grade | I, II/III | 1.031 (0.758-1.402) | 0.847 |
|  | Lymphatic invasion | No/Yes | 1.113 (0.743-1.669) | 0.603 |
|  | Vascular invasion | No/Yes | 1.482 (1.037-2.119) | 0.031 |
|  | HR status | Negative/Positive | 1.128 (0.821-1.550) | 0.457 |
|  |  |  |  |  |
| B. Breast cancer-specific survival | |  |  |  |
| Stage | Factors |  | HRs (95% CI) | *p-*value |
| Stage I | Age (years)* | Per unit | 1.086 (1.030-1.145) | 0.002 |
|  | Grade | I, II/III | 0.655 (0.166-2.576) | 0.545 |
|  | Lymphatic invasion | No/Yes | 3.236 (0.685-15.283) | 0.138 |
|  | Vascular invasion | No/Yes | 0.839 (0.120-5.863) | 0.859 |
|  | HR status | Negative/Positive | 2.781 (0.731-10.586) | 0.134 |
| Stage II | Age (years)* | Per unit | 1.054 (1.021-1.087) | 0.001 |
|  | Grade | I, II/III | 1.514 (0.750-3.057) | 0.248 |
|  | Lymphatic invasion | No/Yes | 2.820 (1.212-6.560) | 0.016 |
|  | Vascular invasion | No/Yes | 1.036 (0.445-2.410) | 0.935 |
|  | HR status | Negative/Positive | 1.977 (0.941-4.151) | 0.072 |
| Stage III | Age (years)* | Per unit | 1.015 (0.983-1.048) | 0.352 |
|  | Grade | I, II/III | 1.192 (0.616-2.308) | 0.601 |
|  | Lymphatic invasion | No/Yes | 1.433 (0.543-3.781) | 0.467 |
|  | Vascular invasion | No/Yes | 1.880 (0.868-4.070) | 0.109 |
|  | HR status | Negative/Positive | 1.214 (0.630-2.340) | 0.562 |
| HRs; hazard ratios, CI; confidence interval, HR: hormonal receptor | | | |  |
| *Continuous variables | |  |  |  |
